# Abnormally Low or High Ankle-Brachial Index Is Associated with Proliferative Diabetic Retinopathy in Type 2 Diabetic Mellitus Patients

**DOI:** 10.1371/journal.pone.0134718

**Published:** 2015-07-31

**Authors:** Szu-Chia Chen, Pi-Jung Hsiao, Jiun-Chi Huang, Kun-Der Lin, Wei-Hao Hsu, Yu-Li Lee, Mei-Yueh Lee, Jer-Ming Chang, Shyi–Jang Shin

**Affiliations:** 1 Division of Nephrology, Department of Internal Medicine, Kaohsiung Medical University Hospital, Kaohsiung Medical University, Kaohsiung, Taiwan; 2 Division of Endocrinology and Metabolism, Department of Internal Medicine, Kaohsiung Medical University Hospital, Kaohsiung Medical University, Kaohsiung, Taiwan; 3 Department of Internal Medicine, Kaohsiung Municipal Hsiao-Kang Hospital, Kaohsiung Medical University, Kaohsiung, Taiwan; 4 Department of Internal Medicine, Kaohsiung Municipal Ta-Tung Hospital, Kaohsiung Medical University, Kaohsiung, Taiwan; 5 Graduate Institute of Clinical Medicine, College of Medicine, Kaohsiung Medical University, Kaohsiung, Taiwan; 6 Faculty of Medicine, College of Medicine, Kaohsiung Medical University, Kaohsiung, Taiwan; 7 Renal Care, College of Medicine, Kaohsiung Medical University, Kaohsiung, Taiwan; 8 Center for Lipid and Glycomedicine Research, Kaohsiung Medical University, Kaohsiung, Taiwan; Temple University School of Medicine, UNITED STATES

## Abstract

Although some studies have reported that low ankle-brachial index (ABI) is associated with diabetic retinopathy (DR) in diabetic patients, it remains controversial as to which stage of DR. The aim of this study is to assess whether peripheral artery disease (PAD), indicated by abnormally low or high ABI, is associated with different stages of DR in patients with type 2 diabetes mellitus (DM), and further evaluate the risk factors. A total of 2001 (858 men and 1143 women) patients with type 2 DM who underwent ABI measurement in an outpatient clinic were enrolled. PAD was defined as ABI < 0.9 or ≧ 1.3 in either leg. DR was classified as non-DR, nonproliferative DR and proliferative DR stages. The clinical data were analyzed and the risk factors for abnormal ABI were determined by multivariate logistic regression analysis. The prevalence of ABI < 0.9 or ≧ 1.3 was 3.0%. Multivariate forward logistic regression analysis identified proliferative DR (*vs*. non-DR) was associated with abnormal ABI (odds ratio, 1.718; 95% confidence interval, 1.152 to 2.562; *p* = 0.008), but nonproliferative DR was not. Furthermore, the presence of coronary artery disease, cerebrovascular disease, declining renal function and patients without diuretics use were associated with abnormal ABI in patients with proliferative DR. Our study in patients of type 2 DM demonstrated that PAD was associated with proliferative DR. We emphasize the recommendation of performing the ABI test in this population at risk.

## Introduction

Diabetes mellitus (DM) is a high risk population for peripheral artery disease (PAD), a major artery disease caused by atherosclerosis [1]. Previous study has reported that more than half of the diabetic patients who underwent an amputation had PAD in Taiwan [2]. The prognosis of diabetic patients undergoing lower extremity amputation is poor with 5-year survival rate about 50% [3]. The ankle-brachial index (ABI) is reported to be a good marker for atherosclerosis, and an ABI < 0.9 is useful in the diagnosis of peripheral artery occlusive disease (PAOD) [4–6]. In addition, an ABI ≧ 1.3 is considered to indicate medial artery calcification (MAC) [7]. High prevalence of PAOD and increased MAC are frequently noted in patients with DM [8, 9]. Moreover, either abnormally low or high ABI can predict cardiovascular morbidity and mortality in patients with DM [10, 11].

Similar to atherosclerosis, diabetic retinopathy (DR) is associated with cardiovascular risk in type 2 diabetic patients [12]. Some epidemiological studies have identified the association between PAD and DR [13, 14, 15], whereas others have failed to identify this relation [16, 17]. Thus, the relationship between PAD and DR remains controversial and whether the relation still exists in different stages of DR is unclear. There are limited studies to evaluate the association of PAD and different stages of DR in diabetic patients. Accordingly, the aim of this study is to assess whether PAD, indicated by abnormally low or high ABI, is associated with different stages of DR in patients with type 2 DM, and further evaluate the risk factors.

## Materials and Methods

### Study Patients

All patients with type 2 DM who visited the diabetic clinic in the Internal Medicine out-patient departments of two hospitals in southern Taiwan between April 2002 and November 2004 were included in the study. We excluded patients with type 1 DM (defined as presentation with diabetic ketoacidosis, acute hyperglycemia symptoms with heavy ketonuria [≧ 3], or the continuous requirement of insulin in the year succeeding diagnosis), patients under dialysis or with estimated glomerular filtration rate (eGFR) less than 15 ml/min/1.73m^2^, and patients who received renal transplantation. Finally, two thousand and one patients (mean age 64.1 ± 11.3 years, 858 males and 1143 females) were included in this study.

### Ethics Statement

The study protocol was approved by the institutional review board of the Kaohsiung Medical University Hospital (KMUHIRB-E-20150029). Informed consents have been obtained in written form from patients and all clinical investigation was conducted according to the principles expressed in the Declaration of Helsinki. The patients gave consent for the publication of the clinical details.

### Assessment of ABI

The values of ABI were measured by using an ABI-form device (VP1000; Colin Co. Ltd., Komaki, Japan), which automatically and simultaneously measured blood pressures in both arms and ankles using an oscillometric method [18–20]. The ABI was calculated by the ratio of the ankle systolic blood pressure divided by the arm systolic blood pressure. The ABI measurement was done once in each patient. PAD was defined as ABI < 0.9 or ≧ 1.3 in either leg. The validation of this automatic device and its reproducibility had been previously published [19].

### Collection of Demographic, Medical, and Laboratory Data

Demographic and medical data including age, gender, and co-morbid conditions were obtained from medical records and interviews with patients. The body mass index (BMI) was calculated as the ratio of weight in kilograms divided by square of height in meters. Laboratory data were measured from fasting blood samples using an autoanalyzer (Roche Diagnostics GmbH, D-68298 Mannheim COBAS Integra 400). Serum creatinine was measured by the compensated Jaffé (kinetic alkaline picrate) method in a Roche/Integra 400 Analyzer (Roche Diagnostics, Mannheim, Germany) using a calibrator traceable to isotope-dilution mass spectrometry [21]. The value of eGFR was calculated using the 4-variable equation in the Modification of Diet in Renal Disease (MDRD) study [22]. Urine albumin and creatinine were measured on a spot urine sample by an autoanalyzer (COBAS Integra 400 plus; Roche Diagnostics, North America), and microalbuminuria was defined as the ratio of urine albumin to creatinine of ≧ 30 mg/gm. Blood samples were obtained within 1 month of ABI measurement. In addition, information regarding patient medications, including angiotensin-converting enzyme inhibitors (ACEIs), angiotensin II receptor blockers (ARBs), β-blockers, calcium channel blockers, diuretics, and HMG-CoA reductase inhibitors (statins), during the study period was obtained from medical records.

### DR Evaluation

DR was evaluated by experienced ophthalmologists while the patients’ pupils were dilated. If needed, fluorescein angiography was performed. DR was classified as non-DR, nonproliferative DR and proliferative DR stages [23].

### Statistical Analysis

Statistical analysis was performed using SPSS 19.0 for windows (SPSS Inc. Chicago, USA). Data are expressed as percentages, mean ± standard deviation, or median (25^th^-75^th^ percentile) for triglyceride. The differences between groups were checked by Chi-square test for categorical variables and by independent t-test for continuous variables. Multiple forward logistic regression analysis after adjustment of age, sex, coronary artery disease, cerebrovascular disease, systolic blood pressures, pulse pressure, BMI, hemoglobin A1c, fasting glucose, log triglyceride, total cholesterol, high density-lipoprotein (HDL)-cholesterol, low density-lipoprotein (LDL)-cholesterol, eGFR, microalbuminuria, diabetic retinopathy stage, anti-hypertensive medications, and statins use was used to identify the factors associated with an abnormal ABI. A difference was considered significant if the *p* value was less than 0.05.

## Results

A total of two thousand and one type 2 DM patients were included. The mean age was 64.1 ± 11.3 years and there were 858 males and 1143 females. The prevalence of ABI < 0.9 or ≧ 1.3 was 3.0% (61/2001). The comparison of baseline characteristics between patients with and without a normal ABI was shown in Table 1. There were 1840 and 61 patients in 2 groups, respectively. Compared with patients with a normal ABI of ≧ 0.9 to < 1.3, patients with an abnormal ABI of < 0.9 or ≧ 1.3 were found to have older age, higher prevalence of coronary artery disease, high prevalence of cerebrovascular disease, lower diastolic blood pressure, higher pulse pressure, higher BMI, higher triglyceride, lower HDL-cholesterol, lower eGFR, higher prevalence of microalbuminuria, more advanced DR stages and higher prevalence of anti-hypertensive medications and statins use.

**Table 1:** Comparison of baseline characteristics between patients with and without a normal ABI of ≧ 0.9 to < 1.3.

Characteristics	All patients (n = 2001)	ABI ≧ 0.9 to < 1.3 (n = 1840)	ABI < 0.9 or ≧ 1.3 (n = 61)	*p*
Age (year)	64.1 ± 11.3	63.6 ± 11.3	68.8 ± 10.6	< 0.001
Male gender (%)	42.9	42.7	44.7	0.632
Coronary artery disease (%)	16.8	15.9	27.3	< 0.001
Cerebrovascular disease (%)	4.9	4.2	13.0	< 0.001
Systolic blood pressure (mmHg)	134.9 ± 18.8	134.9 ± 18.8	135.2 ± 18.5	0.825
Diastolic blood pressure (mmHg)	77.8 ± 11.3	78.0 ± 11.3	75.8 ± 11.7	0.022
Pulse pressure (mmHg)	57.1 ± 15.3	56.9 ± 15.2	59.4 ± 15.5	0.049
Body mass index (kg/m^2^)	25.8 ± 3.6	25.8 ± 3.6	26.5 ±3.5	0.009
Laboratory parameters				
HbA1c (%)	7.7 ± 1.7	7.7 ± 1.7	7.6 ± 1.6	0.722
Fasting glucose (mg/dL)	148.5 ± 51.5	148.6 ± 51.5	147.7 ± 50.8	0.836
Triglyceride (mg/dL)	126 (91–179)	125 (90–176)	139.5 (106.5–214.75)	0.001
Total cholesterol (mg/dL)	185.7 ± 38.7	185.5 ± 38.4	187.7 ± 41.3	0.498
HDL-cholesterol (mg/dL)	49.5 ± 13.1	49.7 ± 13.1	47.2 ± 12.5	0.020
LDL-cholesterol (mg/dL)	104.3 ± 28.5	104.3 ± 28.4	104.6 ± 29.5	0.882
eGFR (mL/min/1.73 m^2^)	68.7 ± 19.7	69.4 ± 19.4	60.2 ± 20.4	< 0.001
Microalbuminuria (%)	34.9	33.8	47.2	0.001
DR stage				0.001
Non-DR (%)	65.0	66.0	53.4	
Nonproliferative DR (%)	17.0	16.8	18.6	
Proliferative DR (%)	18.0	17.2	28.0	
Medications				
ACEI and/or ARB use (%)	73.3	72.6	84.5	0.001
β-blocker use (%)	23.4	22.6	31.7	0.009
Calcium channel blocker use (%)	39.2	37.6	57.1	< 0.001
Diuretic use (%)	46.0	44.8	59.0	0.001
Statins use (%)	59.9	59.1	65.3	0.023

Abbreviations. ABI, ankle-brachial index; HbA1c, hemoglobin A1c; HDL, high-density lipoprotein; LDL, low-density lipoprotein; eGFR, estimated glomerular filtration rate; DR, diabetic retinopathy; ACEI, angiotensin converting enzyme inhibitor; ARB, angiotensin II receptor blocker.

### Determinants of Abnormal ABI in Total Patients

Table 2 presented the determinants of abnormal ABI in total patients. In the multivariate forward logistic regression analysis after adjusting for age, sex, coronary artery disease, cerebrovascular disease, systolic blood pressures, pulse pressure, BMI, hemoglobin A1c (HbA1c), fasting glucose, log triglyceride, total cholesterol, HDL-cholesterol, LDL-cholesterol, eGFR, microalbuminuria, DR stages, anti-hypertensive medications and statins use, proliferative DR was independently associated with abnormal ABI (*vs*. non-DR; odds ratio [OR], 1.718; 95% confidence interval [CI], 1.152 to 2.562; *p* = 0.008), but nonproliferative DR was not (*vs*. non-DR; OR, 1.190; 95% CI, 0.763 to 7.856; *p* = 0.442). In addition, age (per 1 year; OR, 1.035; 95% CI, 1.016 to 1.054; *p* < 0.001), cerebrovascular disease (OR, 2.200; 95% CI, 1.281 to 3.779; *p* = 0.004), triglyceride (log per 1 mg/dL; OR, 2.376; 95% CI, 1.163 to 4.852; *p* = 0.018) and eGFR (per 1 mL/min/1.73 m^2^; OR, 0.989; 95% CI, 0.979 to 0.999; *p* = 0.028) were independently associated with abnormal ABI.

**Table 2:** Determinants of abnormal ABI using multivariate logistic regression analysis in total patients.

Parameter	Multivariate (forward)	
	OR (95% CI)	*p*
Age (per 1 year)	1.035 (1.016–1.054)	< 0.001
Cerebrovascular disease	2.200 (1.281–3.779)	0.004
Triglyceride (log per 1 mg/dL)	2.376 (1.163–4.852)	0.018
eGFR (per 1 mL/min/1.73 m^2^)	9.989 (0.979–0.999)	0.028
DR stage		
Non-DR	Reference	
Nonproliferative DR	1.190 (0.763–1.856)	0.442
Proliferative DR	1.718 (1.152–2.562)	0.008

Values expressed as odds ratio (OR) and 95% confidence interval (CI). Adjusted for age, sex, coronary artery disease, cerebrovascular disease, systolic blood pressures, pulse pressure, body mass index, HbA1c, fasting glucose, log triglyceride, total cholesterol, HDL-cholesterol, LDL-cholesterol, eGFR, microalbuminuria, diabetic retinopathy stage, anti-hypertensive medications and statins use. Abbreviations. ABI, ankle-brachial index; HbA1c, hemoglobin A1c; HDL, high-density lipoprotein; LDL, low-density lipoprotein; eGFR, estimated glomerular filtration rate; DR, diabetic retinopathy; ACEI, angiotensin converting enzyme inhibitor; ARB, angiotensin II receptor blocker.

Because serum glucose control might affect the progression of DR, we further evaluated the association of DR stage and abnormal ABI in patients with HbA1C ≦ 7% (n = 837). After multiple logistic regression analysis, we found that proliferative DR was still independently associated with abnormal ABI (*vs*. non-DR; OR, 2.473; 95% CI, 1.255 to 4.872; *p* = 0.009), but nonproliferative DR was not (*vs*. non-DR; OR, 1.882; 95% CI, 0.981 to 3.610; *p* = 0.057).

### Determinants of Abnormal ABI in Patients with Proliferative DR

Table 3 presented the determinants of abnormal ABI in patients with proliferative DR. In the multivariate forward logistic regression analysis after adjusting for age, sex, coronary artery disease, cerebrovascular disease, systolic blood pressures, pulse pressure, BMI, HbA1c, fasting glucose, log triglyceride, total cholesterol, HDL-cholesterol, LDL-cholesterol, eGFR, microalbuminuria, anti-hypertensive medications and statins use, coronary artery disease (OR, 2.508; 95% CI, 1.210 to 5.199; *p* = 0.013), cerebrovascular disease (OR, 3.057; 95% CI, 1.239 to 7.543; *p* = 0.015), eGFR (per 1 mL/min/1.73 m^2^; OR, 0.963; 95% CI, 0.944 to 0.982; *p* < 0.001) and diuretics use (OR, 0.409; 95% CI, 0.200 to 0.837; *p* = 0.014) were independently associated with an abnormal ABI.

**Table 3:** Determinants of abnormal ABI using multivariate logistic regression analysis in patients with proliferative diabetic retinopathy.

Parameter	Multivariate (forward)	
	OR (95% CI)	*p*
Coronary artery disease	2.508 (1.210–5.199)	0.013
Cerebrovascular disease	3.057 (1.239–7.543)	0.015
eGFR (per 1 mL/min/1.73 m^2^)	0.963 (0.944–0.982)	< 0.001
Diuretics use	0.409 (0.200–0.837)	0.014

Values expressed as odds ratio (OR) and 95% confidence interval (CI). Adjusted for age, sex, coronary artery disease, cerebrovascular disease, systolic blood pressures, pulse pressure, body mass index, HbA1c, fasting glucose, log triglyceride, total cholesterol, HDL-cholesterol, LDL-cholesterol, eGFR, microalbuminuria, anti-hypertensive medications and statins use. Abbreviations. ABI, ankle-brachial index; HbA1c, hemoglobin A1c; HDL, high-density lipoprotein; LDL, low-density lipoprotein; eGFR, estimated glomerular filtration rate; DR, diabetic retinopathy; ACEI, angiotensin converting enzyme inhibitor; ARB, angiotensin II receptor blocker.

### Reproducibility

The mean percent error for ABI measurement was 3.58 ± 3.15%.

## Discussion

In the present study, we evaluated the association of PAD and DR in patients with type 2 DM. We found that ABI < 0.9 or ≧ 1.3 was associated with proliferative DR, but not nonproliferative DR. Besides, old age, cerebrovasculat disease, hypertriglyceridemia and declining renal function were associated with abnormal ABI in total patients. Furthermore, the presence of coronary artery disease, cerebrovascular disease, declining renal function and patients without diuretics use were risk factors for abnormal ABI in patients with proliferative DR.

A clinical device, the ABI-form (Colin VP1000, Komaki, Japan), has been developed to automatically and simultaneously measure blood pressures in both arms and ankles. The value of ABI, which is a good marker for atherosclerosis, can be obtained easily with this device [19, 24]. Low ABI is reported to be associated with generalized atherosclerosis (e.g. common carotid artery intima-media thickness and the degree of stenosis in the intracranial internal carotid artery and middle cerebral artery) [25, 26]. However, in diabetic patients, it has some specific caveats. Although low ABI is very common in diabetic patients, PAD prevalence remains under-estimated. Sensitivity of the standard threshold of 0.9 appears to be lower in diabetic patients. The decrease in ABI sensitivity can be explained by arterial stiffness secondary to MAC [27]. There is strong association between diabetes and MAC [8,9]. Falsely elevated pressures or incompressible arteries at ankle level are common among diabetic patients with extensive vascular calcification of the lower extremities [7, 28]. Moreover, patients having abnormally high ABI also have poor prognosis for all-cause and cardiovascular mortality in diabetic patients [10, 11]. High ABI values in diabetes could be indicative of PAD. Therefore, the presence of a PAD is not only defined by ABI < 0.9, but also with values ≧ 1.3 in our present study.

The important finding of our study is the identification of the association between abnormally low or high ABI with proliferative DR. Yu JH et al. had evaluated the risk factors of PAD, and found DR was associated with ABI ≦ 0.9 in 2002 Korean patients with type 2 DM [14]. Li X et al. also found that DR was related with ABI < 0.9, but not with ABI > 1.3 in 3924 diabetic patients [13]. Chen YW et al. had compared the prevalence of PAD in type 2 DM patients with non-DR and proliferative DR using ABI, toe-brachial index and duplex ultrasound. They found PAD is more common in proliferative DR than in non-DR [15]. The exact mechanisms underlying the association between atherosclerosis and DR are poorly understood. Some studies have evaluated the association, and one possible explanation is that atherosclerosis and DR share common risk factors in the causal pathway [29, 30]. Such evidence is supported by a “common mechanism” for the development of macro- and microangiography in type 2 DM, such as obesity, insulin-resistance and hypertension [31, 32]. However, in previous studies, patients were considered to have retinopathy if they displayed the nonproliferative or proliferative stages [13, 14]. In our study, we divided the study patients into non-DR, nonproliferative DR and proliferative DR stages, respectively, and further found that abnormal ABI was correlated with proliferative DR, not nonproliferative DR. Hence, assessment of ABI in diabetic patients with proliferative DR may help identify the high-risk group with PAD.

The control of serum glucose levels has been associated with the progression and severity of DR [33, 34]. In Chen YW’s study, they excluded type 2 DM patients with HbA1c < 7.5%, and found PAD is associated to proliferative DR [15]. In this study, we further evaluated the association of DR stage and abnormal ABI in type 2 DM patients with HbA1C ≦ 7%, and still found proliferative DR was still independently associated with abnormal ABI. Previous study has also shown that DR is present in persons with elevated fasting glucose and impaired glucose tolerance and no known history of diabetes [35]. These findings imply that retinopathy may occur over a wider continuum of glycemia. Therefore, earlier screening for retinopathy and ABI in the pre-diabetic state and glucose control well patients is suggested.

In the present study, we found that declining eGFR was associated with PAD in regardless total or proliferative DR patients. Decreased eGFR may predispose to increased atherosclerosis with multiple pathogenic mechanisms involved, including deranged calcium/phosphate balance, secondary hyperparathyroidism, homocysteine, lipoprotein(a) metabolism, fluid overload, alterations in the angiotensin and endothelin systems, malnutrition, oxidative stress, insulin resistance, and alterations in inflammatory and coagulation pathways [36, 37]. Therefore, when evaluating PAD, renal function must be considered.

Another finding of our study is the identification of the association between abnormally low or high ABI with hypertriglyceridemia. Some studies have shown that triglyceride level is a predictive factor for PAD in diabetic patients [14, 38], though not all [39, 40]. Hypertriglyceridemia is known to be related with increased levels of prothrombotic factors, such as fibrinogen and plasminogen activator inhibitors, and is associated to the size and density of LDL particle [41, 42]. In addition, hypertriglyceridemia is associated with some atherogenic remnant particles, and apo C-III [43]. This may explain our findings that hypertriglyceridemia is a risk factor for PAD in diabetes.

Our study had several limitations. First, this was a cross-sectional study. The association between abnormal ABI and proliferative DR cannot be taken as a causal relationship. Nonetheless, the result may help shed light on the importance of the ABI test in this population at risk. Further prospective studies are needed to evaluate the development and progression of macro- and microangiography in diabetes. In addition, only ABI measurement was done once in each patient, which could cause bias data. Finally, the majority of our patients were treated chronically with antihypertensive medications. For ethical reasons, we did not withdraw these medications. Hence, we could not exclude the influence of the aforementioned medications on our findings. However, to minimize the influence of drugs, we included medication use in the multivariate analysis.

In conclusion, our results demonstrated that PAD was associated with proliferative DR in patients with type 2 DM. We emphasize the recommendation of performing the ABI test in this population at risk.

## Supporting Information

S1 Dataset Relevant data including DR stage and ABI. (XLS)
